# Long-Term Outcome Following Coronary Artery Stenting by History of Preterm Delivery

**DOI:** 10.1016/j.jacadv.2022.100142

**Published:** 2022-11-30

**Authors:** Moa Pehrson, Andreas Edsfeldt, Giovanna Sarno, Abigail Fraser, Janet W. Rich-Edwards, Mats Pihlsgård, Simon Timpka

**Affiliations:** a Perinatal and Cardiovascular Epidemiology, Department of Clinical Sciences Malmö, Lund University, Malmö, Sweden; b Cardiovascular Research- Translational Studies, Lund University, Malmö, Sweden; c Wallenberg Center for Molecular Medicine, Lund University, Lund, Sweden; d Department of Cardiology, Skåne University Hospital, Lund/Malmö, Sweden; e Department of Medical Sciences, Cardiology and Uppsala Clinical Research Center, Uppsala University, Uppsala, Sweden; f Population Health Science, Bristol Medical School, University of Bristol, Bristol, UK; g Division of Women’s Health, Department of Medicine, Brigham and Women’s Hospital and Harvard Medical School, Boston, Massachusetts, USA; h Department of Obstetrics and Gynecology, Skåne University Hospital, Malmö, Sweden

**Keywords:** major adverse cardiovascular events, percutaneous coronary intervention, preterm delivery

## Abstract

**Background:**

Women are at a greater risk of a major adverse cardiovascular event (MACE) after percutaneous coronary intervention than men. A history of preterm delivery is a female-specific risk factor for coronary artery disease, but its relevance in the treatment of coronary artery disease is unknown.

**Objectives:**

The purpose of this study was to analyze the association between a history of preterm delivery and MACE after the first coronary artery stenting.

**Methods:**

We included a nationwide sample of 5,766 Swedish women aged ≤65 years receiving their first coronary stent during 2006 to 2017. To adjust for periprocedural characteristics and estimate the association between a history of preterm delivery and MACE at >30 days from stenting, we used proportional hazards regression. We also investigated mortality by history of preterm delivery.

**Results:**

During a median follow-up time of 3.7 years (IQR: 1.3-6.7 years), 1,200 (20.8%) women had a MACE. In total, 963 (16.7%) women had a history of preterm delivery. A history of preterm delivery was associated with a higher risk of MACE (adjusted HR: 1.19; 95% CI: 1.03-1.38) and mortality (adjusted HR ratio: 1.38; 95% CI: 1.02-1.85). Similar associations were observed when excluding women with a history of hypertensive disorders of pregnancy or diabetes. Subgroup analyses suggested that women with a history of early preterm delivery had lower risk of MACE than those who had late preterm delivery (*P* = 0.04).

**Conclusions:**

History of preterm delivery is associated with worse prognosis following the first coronary artery stenting in women and warrants consideration as a risk factor also in the secondary prevention setting.

Cardiovascular disease (CVD) is the most common cause of death in women.^1^ Female sex is also associated with major adverse cardiovascular events (MACEs)^2^^,^^3^ and worse prognosis after a percutaneous coronary intervention (PCI).^4^ Although women undergoing a PCI might have a higher comorbidity burden than men undergoing the procedure,^3^ female-specific prognostic factors have rarely been investigated in this context.^5^, ^6^, ^7^ Women with a history of preterm delivery are more likely to experience a cardiovascular event or a cardiovascular-related death than other women.^8^, ^9^, ^10^ Several other key risk factors for developing symptomatic coronary artery disease (CAD), such as older age, diabetes mellitus, hypertension, and smoking, are also predictors of worse outcomes following PCI.^11^^,^^12^ To our knowledge, the relevance of a history of preterm delivery as a prognostic factor following treatment with coronary artery stenting, ie, in the secondary prevention setting, is unknown.

To address this, we studied the association between a history of preterm delivery and clinical outcomes after coronary artery stenting using data from comprehensive Swedish registers of deliveries and PCIs. We hypothesized that women with a history of preterm delivery would have increased risk of MACE following coronary artery stenting.

## Methods

**Figure 1:**
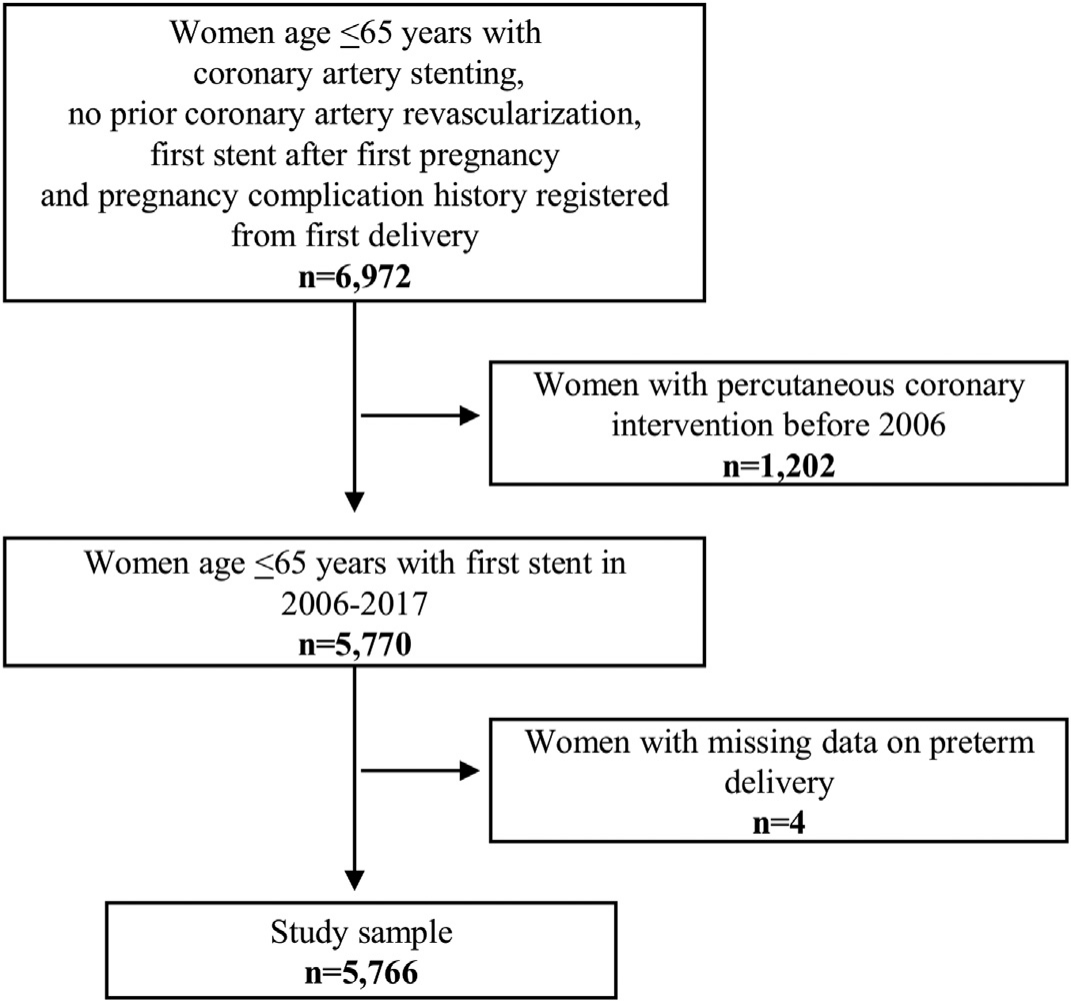
**Flowchart of Study Sample** The figure shows the inclusion and exclusion criteria for the study sample.

Study data originated from 2 comprehensive Swedish registers: SCAAR (Swedish Coronary Angiography and Angioplasty Registry) and the Medical Birth Register (MBR) from the National Board of Health and Welfare. Women were included if they had a coronary stenting procedure recorded in the SCAAR during 2006 to 2017 after their first delivery, and their first delivery was recorded in the MBR (Figure 1). Deliveries recorded after the first coronary stenting procedure were not included in the study. We excluded women who underwent PCI procedures before 2006 as data on all covariates were not routinely collected prior to 2006. To identify incident events during follow-up, we used 3 additional registers: the Register of Information and Knowledge About Swedish Heart Intensive Care, the Swedish National In-patient Register, and the Swedish National Death Register. Data on patients lost at follow-up due to emigration were obtained from Statistics Sweden. Information was linked using the Swedish personal identity number.^13^ The study was approved by the Ethical Review Board in Lund, Sweden.

### Index coronary artery stenting procedure

SCAAR has previously been described.^14^, ^15^, ^16^ In short, it is a comprehensive procedure-related register aiming to document information on all coronary angiographies and PCI procedures in Sweden. Each procedure in SCAAR is described with angiographic, demographic, and procedure-related variables. We grouped coronary segments identified in SCAAR into specific vessels treated: left main stem (LMS), left anterior descending artery (LAD), right coronary artery, left circumflex coronary artery, and other. We further combined treated LMS and LAD into 1 category due to few observations in the LMS group. The indication for coronary artery stenting was categorized as ST-segment elevation myocardial infarction (MI), non-ST-segment elevation MI, unstable CAD, stable CAD, and others (eg, cardiac arrest, heart failure, unclear chest pain). Multiple-vessel disease is defined as women could present with findings in more than 1 coronary artery. To account for general improvement of care during the study period, we categorized the year of procedure into 3 categories: 2006 to 2009, 2010 to 2013, and 2014 to 2017. In addition to the procedure-specific variables mentioned above, SCAAR also records clinical characteristics of individuals included in the register at the time of PCI. Women with hypertension and/or dyslipidemia were identified based on receiving antihypertensive treatment or lipid-lowering agents at the time of coronary artery stenting, respectively. Diabetes was identified as a known diabetes diagnosis at the time of coronary artery stenting, regardless of treatment. Smoking status was divided into the categories such as never smoker, ex-smoker, or current smoker at the time of coronary artery stenting. Prior MI was defined by SCAAR as MI prior to the current hospitalization, including silent MI based on electrocardiography or echocardiography findings.

### Preterm delivery and pregnancy history

The MBR is a Swedish register which has collected data on almost all deliveries in Sweden since 1973.^17^ To ascertain the pregnancy history of women included in the study, we included women with their first delivery registered in MBR. We defined preterm delivery as delivery before 37 + 0 weeks of gestation. Preterm delivery history was further defined according to the woman’s most preterm delivery prior to her first coronary artery stenting. For subgroup analyses, we further classified preterm delivery into 2 categories namely late preterm delivery (34 + 0-36 + 6 weeks of gestation) and early preterm delivery (22 + 0-33 + 6 weeks of gestation).

### Clinical endpoints

Our primary endpoint was MACE defined as either an incident cardiovascular event or a cardiovascular death at >30 days from coronary artery stenting. See Supplemental Table 1 for a full list of diagnoses according to the International Classification of Diseases. Our secondary objective was to examine mortality after coronary artery stenting by history of preterm delivery, defined as death at >30 days from the index coronary artery stenting procedure. We included outcomes at >30 days after the coronary artery stenting to minimize the risk of capturing the patient’s primary event.

### Statistical analysis

We summarized characteristics of the study sample as means or percentages and calculated the percentages of missing data for each variable. For graphic visualization, event rates were estimated using the Kaplan-Meier method and comparisons made using the log-rank test.

To study preterm delivery as a prognostic marker of MACE, we used proportional hazard regression. Right censoring during follow-up occurred at the end of follow-up in 2017, during migration out of Sweden, or due to death not related to MACE during follow-up, whichever came first. We adjusted for possible prognostic factors in 3 steps. In model I, we included preterm delivery history and age. In model II, we additionally adjusted for procedure type, indication for coronary artery stenting, year of procedure, number of vessels treated, number of stents, multiple vessel disease, drug-eluting stent, LMS or LAD treated, right coronary artery treated, left circumflex coronary artery treated, and any other vessel treated. In model III, we further adjusted for diabetes, smoking, hypertension, dyslipidemia, and prior MI.

To investigate preterm delivery as a prognostic marker of long-term mortality, we also used proportional hazards regression. Right censoring during follow-up occurred during migration out of Sweden or at the end of follow-up in 2017, whichever came first. We adjusted the analysis on mortality for prognostic factors as described above for MACE.

To determine whether any association may be driven by hypertensive disorders of pregnancy (HDPs) or diabetes mellitus at the time of procedure, secondary analyses restricted to women without a history of HDP and without diabetes mellitus were also performed. As additional analyses, we repeated the analyses with severity of preterm delivery (history of late or early preterm delivery) as the exposure.

A small minority of individuals (n = 293) had missing data on any covariable, and we used multiple imputation to impute these missing data. Twenty imputed data sets were created using multiple imputation by chained equations, and data were analyzed with the command mi estimate. We repeated all major analyses using a complete case data set in which individuals with missing data were excluded. The proportional hazards assumption was assessed graphically. Statistical analyses were conducted using Stata 16.0 (StataCorp LLC). A significance level of *P* < 0.05 was used for hypothesis testing.

## Results

Table 1 shows the characteristics of the study sample (N = 5,766) at the time of the first coronary artery stenting by history of preterm delivery. In total, 963 (16.7%) women had a history of preterm delivery. Of these, 652 (11.3%) women had a history of late preterm delivery, and 311 (5.4%) women had a history of early preterm delivery. Women with a history of preterm delivery more often presented with diabetes, hypertension, and dyslipidemia at the time of stenting than other women. They were also more likely to have a history of prior MI.

**Table 1:** Clinical Characteristics of Study Sample by History of Preterm Delivery (N = 5,766)

	No Preterm Delivery (n = 4,803)	Missing	Ever Preterm Delivery (n = 963)	Missing	Subgroups of Preterm Delivery (n = 963)	
					Late Preterm Delivery (n = 652)	Early Preterm Delivery (n = 311)
Age, y	55.3 ± 6.4	-	53.9 ± 6.9	-	54.4 ± 6.6	52.9 ± 7.3
Diabetes mellitus	673 (14.1%)	19 (0.4)	225 (23.5%)	4 (0.4)	149 (23.0%)	76 (24.5%)
Current smoker	2,145 (46.1%)	146 (3.0)	458 (49.3%)	33 (3.4)	305 (48.3%)	153 (51.2%)
Hypertension	2,094 (44.2%)	63 (1.3)	451 (47.4%)	12 (1.2)	306 (47.7%)	145 (46.9%)
Dyslipidemia	1,412 (29.9%)	78 (1.6)	340 (35.8%)	14 (1.5)	236 (36.8%)	104 (33.8%)
Prior MI	168 (3.5%)	59 (1.2)	54 (5.7%)	10 (1.0)	32 (5.0%)	22 (7.1%)
Indication for coronary artery stenting						
STEMI	1,743 (36.3%)	-	341 (35.4%)	-	225 (34.5%)	116 (37.3%)
NSTEMI	657 (13.7%)	-	126 (13.1%)	-	85 (13.0%)	41 (13.2%)
Unstable CAD	1,571 (32.7%)	-	311 (32.3%)	-	220 (33.7%)	91 (29.3%)
Stable CAD	697 (14.5%)	-	148 (15.4%)	-	103 (15.8%)	45 (14.5%)
Other	135 (2.8%)	-	37 (3.8%)	-	19 (2.9%)	18 (5.8%)

Values are mean ± SD or n (%).

CAD = coronary artery disease; MI = myocardial infarction; NSTEMI = non-ST-segment elevation myocardial infarction; STEMI = ST-segment elevation myocardial infarction.

Table 2 shows information on the procedure-specific variables by history of preterm delivery. Overall, these characteristics were similar between groups. ST-elevation MI was the most common indication for coronary artery stenting, and LAD (also including LMS) was the vessel most often treated. Women with a history of late preterm delivery were more likely to present with a multiple-vessel disease.

**Table 2:** Procedural Characteristics by History of Preterm Delivery (N = 5,766)

	No Preterm Delivery (n = 4,803)	Ever Preterm Delivery (n = 963)	Subgroups of Preterm Delivery (n = 963)	
			Late Preterm Delivery (n = 652)	Early Preterm Delivery (n = 311)
Vessel treated				
LAD (including LMS)	2,602 (54.2%)	501 (52.0%)	351 (53.8%)	150 (48.2%)
RCA	1,773 (36.9%)	375 (38.9%)	251 (38.5%)	124 (39.9%)
LCX	1,026 (21.4%)	220 (22.9%)	161 (24.7%)	59 (19.0%)
Other	184 (3.8%)	46 (4.8%)	23 (3.5%)	23 (7.4%)
Stent type				
DES^a^	3,197 (69.4%)	677 (72.0%)	458 (71.9%)	219 (72.3%)
Multiple vessel disease^a^				
Yes	1,444 (30.1%)	329 (34.2%)	232 (35.6%)	97 (31.3%)
No of vessels treated				
1	4,098 (85.3%)	805 (83.6%)	535 (82.1%)	270 (86.8%)
≥2	705 (14.7%)	158 (16.4%)	117 (17.9%)	41 (13.2%)
No of stents				
1	3,237 (67.4%)	608 (63.1%)	407 (62.4%)	201 (64.6%)
2	1,058 (22.0%)	239 (24.8%)	154 (23.6%)	85 (27.3%)
≥3	508 (10.6%)	116 (12.1%)	91 (14.0%)	25 (8.0%)
Procedure type				
PCI ad hoc	4,645 (96.7%)	919 (95.4%)	621 (95.3%)	298 (95.8%)
PCI	158 (3.3%)	44 (4.6%)	31 (4.8%)	13 (4.2%)
Year of procedure				
2006-2009	1,288 (26.8%)	230 (23.9%)	153 (23.5%)	77 (24.8%)
2010-2013	1,617 (33.7%)	336 (34.9%)	230 (35.3%)	106 (34.1%)
2014-2017	1,898 (39.5%)	397 (41.2%)	269 (41.3%)	128 (41.2%)

Values are n (%).

DES = drug-eluting stent; LAD = left anterior descending artery; LCX = left circumflex artery; LMS = left main stem; PCI = percutaneous coronary intervention; RCA = right coronary artery

a Multiple-vessel disease, 2 (0.04%) missing in the no preterm delivery group and 1 (0.1%) missing in the ever preterm delivery group; DES, 197 missing (4.1%) in the no preterm delivery group and 23 (2.4%) missing in the ever preterm delivery group.

### MACE following coronary artery stenting by history of preterm delivery

As shown in the Central Illustration, women with a history of preterm delivery had an increased unadjusted rate of MACE. The median follow-up duration was 3.69 years (IQR: 1.43-6.74 years). Women with a history of preterm delivery had a median follow-up duration of 3.35 years (IQR: 1.31-6.23), and women with no history of preterm delivery had a median follow-up duration of 3.77 years (IQR: 1.46-6.86 years).

**Figure 2:**
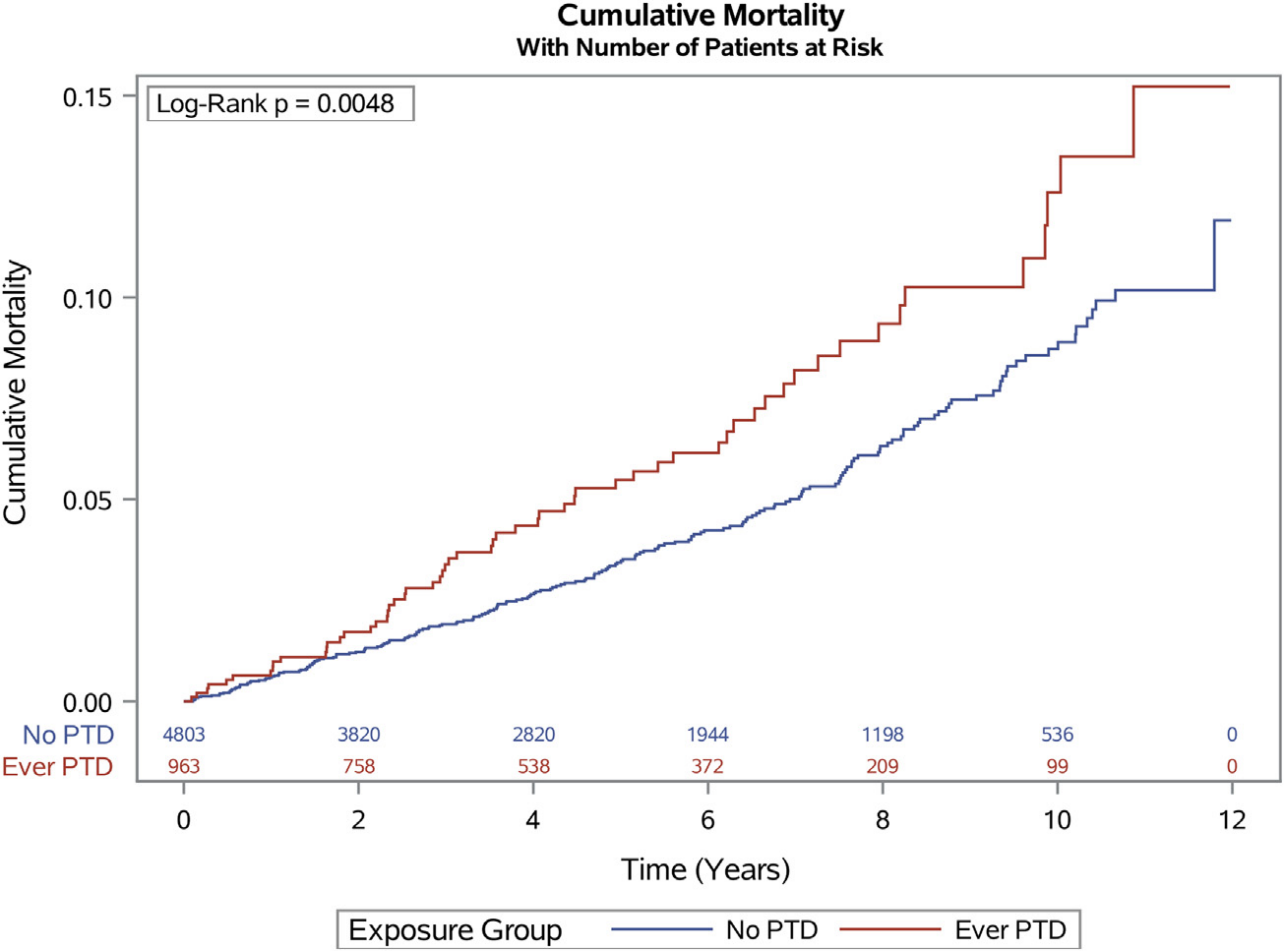
**Cumulative Mortality by History of Preterm Delivery** The figure shows the unadjusted mortality rate by history of preterm delivery following coronary artery stenting. Event rates were estimated using the Kaplan-Meier method, and comparisons were made using the log-rank test. PTD = preterm delivery.

**Central Illustration:**
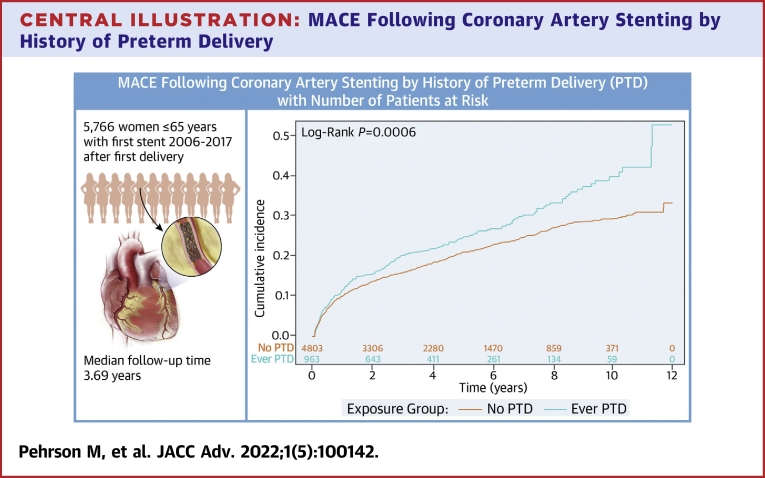
**MACE Following Coronary Artery Stenting by History of Preterm Delivery** A total of 5,766 women aged ≤65 years with first stent placed during 2006 to 2017 after the first delivery—median follow-up duration: 3.69 years. MACE = major adverse cardiovascular events; PTD = preterm delivery.

Table 3 shows the results from the proportional hazards regression analysis on incident MACE following coronary artery stenting by history of preterm delivery. In total, 236 (24.5%) women with a history of preterm delivery experienced a MACE during follow-up, compared to 964 (20%) women with no history of preterm delivery. A history of preterm delivery was associated with higher risk of MACE (adjusted HR: 1.19; 95% CI: 1.03-1.38). Results were similar in the complete case analysis (Supplemental Table 2). Results were also similar in secondary analyses restricted to women without a history of HDP (Supplemental Table 3) and women without diabetes mellitus at the time of the index coronary stenting (Supplemental Table 4). While late preterm delivery was associated with MACE when separately defined in a subgroup analysis (adjusted HR: 1.31; 95% CI: 1.11-1.55), the estimate for early preterm delivery was lower than that for late preterm delivery (*P* = 0.04) and close to 1 (adjusted HR: 0.97; 95% CI: 0.75-1.24). We also preformed 2 sensitivity analyses adjusting our main modes for antithrombotic treatment before coronary artery stenting and during coronary artery stenting. Results were very similar to the corresponding results presented above (data not shown).

**Table 3:** MACE Following Coronary Artery Stenting by Preterm Delivery (N = 5,766)

Preterm Delivery History Events/Person Years	No Preterm Delivery 964/21,089	Ever Preterm Delivery 236/3,902		Subgroups of Preterm Delivery			
				(n = 963)			
				Late Preterm Delivery 169/2,552		Early Preterm Delivery 67/1,350	
		HR (95% CI)	*P* Value	HR (95% CI)	*P* Value	HR (95% CI)	*P* Value
Model I	1.00 (reference)	1.27 (1.10-1.46)	0.001	1.38 (1.17-1.62)	<0.001	1.05 (0.82-1.35)	0.70
Model II	1.00 (reference)	1.25 (1.08-1.45)	0.002	1.36 (1.15-1.60)	<0.001	1.04 (0.81-1.34)	0.75
Model III	1.00 (reference)	1.19 (1.03-1.38)	0.02	1.31 (1.11-1.55)	0.001	0.97 (0.75-1.24)	0.79

Model I includes a history of preterm delivery; age at the index coronary artery stenting (continuous). Model II additionally includes procedure type (PCI, PCI ad hoc); indication for coronary artery stenting (STEMI, NSTEMI, unstable coronary artery disease, stable coronary artery disease, other); year of procedure (2006-2009, 2010-2013, 2014-2017); number of vessels treated (1, ≥2); number of stents (1, 2, ≥3); multiple vessel disease (yes/no); drug-eluting stent (yes/no); left main stem treated or left anterior descending artery treated (yes/no); right coronary artery treated (yes/no); left circumflex coronary artery treated (yes/no); any other vessel treated (yes/no). Model III additionally includes diabetes (yes/no); smoking (never smoker, ex-smoker, current smoker); hypertension (yes/no); dyslipidemia (yes/no); prior MI (yes/no). Results from a proportional hazards regression, multiple imputation analysis. Median follow-up duration: 3.69 years (IQR: 1.43-6.74 years).

CI = confidence interval; HR = hazard ratio; MACE = major adverse cardiovascular events; MI = myocardial infarction; NSTEMI = non-ST-segment elevation myocardial infarction; PCI = percutaneous coronary intervention; STEMI = ST-segment elevation myocardial infarction.

### Long-term mortality following coronary artery stenting by history of preterm delivery

Women with a history of preterm delivery had an increased unadjusted rate of long-term mortality (Figure 2). The median follow-up duration was in total 4.90 years (IQR: 2.42-7.93 years). Women with a history of preterm delivery had a median follow-up duration of 4.70 years (IQR: 2.34-7.62), and women with no history of preterm delivery had a median follow-up duration of 4.95 years (IQR: 2.44-7.99).

Table 4 shows the results from the proportional hazards regression analysis on long-term mortality following coronary artery stenting by history of preterm delivery. Fifty-nine (6.1%) women with a history of preterm delivery died during follow-up, compared to 204 (4.2%) women with no history of preterm delivery. History of preterm delivery was associated with a higher risk of long-term mortality in the crude analysis (model I) for both subgroups of preterm delivery. In the fully adjusted model (model III), only ever preterm delivery remained a prognostic marker of long-term mortality (adjusted HR: 1.38; 95% CI: 1.02-1.85). Results were similar in the complete case analysis (Supplemental Table 5). Supplemental Table 6 shows results from the secondary analysis restricted to women without a history of HDP, and Supplemental Table 7 shows results from the secondary analysis restricted to women without diabetes mellitus at the time of the index stenting procedure. In both secondary analyses, ever preterm delivery remained a prognostic marker of long-term mortality in the fully adjusted model (model III).

**Table 4:** Long-Term Mortality Following Coronary Artery Stenting by Preterm Delivery (N = 5,766)

Preterm Delivery History Events/Person Years	No Preterm Delivery 204/25,443	Ever Preterm Delivery 59/4,916		Subgroups of Preterm Delivery			
				(n = 963)			
				Late Preterm Delivery 38/3,305		Early Preterm Delivery 21/1,611	
		HR (95% CI)	*P* Value	HR (95% CI)	*P* Value	HR (95% CI)	*P* Value
Model I	1.00 (reference)	1.63 (1.22-2.18)	0.001	1.52 (1.08-2.15)	0.02	1.88 (1.20-2.95)	0.006
Model II	1.00 (reference)	1.59 (1.19-2.14)	0.002	1.50 (1.06-2.13)	0.02	1.80 (1.14-2.84)	0.01
Model III	1.00 (reference)	1.38 (1.02-1.85)	0.04	1.30 (0.92-1.85)	0.14	1.53 (0.97-2.43)	0.07

Model I includes a history of preterm delivery; age at the index coronary artery stenting (continuous). Model II additionally includes procedure type (PCI, PCI ad hoc); indication for coronary artery stenting (STEMI, NSTEMI, unstable coronary artery disease, stable coronary artery disease, other); year of procedure (2006-2009, 2010-2013, 2014-2017); number of vessels treated (1, ≥2); number of stents (1, 2, ≥3); multiple vessel disease (yes/no); drug-eluting stent (yes/no); left main stem treated or left anterior descending artery treated (yes/no); right coronary artery treated (yes/no); left circumflex coronary artery treated (yes/no); any other vessel treated (yes/no). Model III additionally includes diabetes (yes/no); smoking (never smoker, ex-smoker, current smoker); hypertension (yes/no); dyslipidemia (yes/no); prior MI (yes/no). Results from a proportional hazards regression, multiple imputation analysis. Median follow-up duration: 4.90 years (IQR: 2.42-7.93 years).

CI = confidence interval; HR = hazard ratio; MI = myocardial infarction; NSTEMI = non-ST-segment elevation myocardial infarction; PCI = percutaneous coronary intervention; STEMI = ST-segment elevation myocardial infarction.

## Discussion

Our key finding is that a history of preterm delivery is associated with 20% increased risk of MACE following coronary stenting in women aged 65 years or younger, an association that remained after adjusting for known predictors of worse outcomes. In further support to consider preterm delivery history in the setting of coronary artery stenting, we also found a history of preterm delivery to be associated with long-term mortality after coronary stenting.

### HDP and diabetes mellitus as potential explanatory factors

Placenta-related complications are a heterogenic group of pregnancy complications associated with a higher rate of mortality following later coronary revascularization.^18^ A major subgroup of these placental disorders is HDP, typically hypertension in pregnancy or preeclampsia, which also is a common cause of iatrogenic preterm delivery. However, as the analyses in which we excluded women with a history of HDP resulted in similar estimates as our main analysis, history of HDP does not seem to explain our reported results. Another potential explanatory factor we considered was diabetes mellitus, which not only is more prevalent in women with a history of preterm delivery^19^ but also increases the risk of adverse outcomes following a PCI.^12^^,^^20^ However, we adjusted for diabetes mellitus in our main analyses, and the estimates were also very similar when we excluded all individuals with diabetes mellitus in a sensitivity analysis.

### Subgroups of preterm delivery

The prevalence of preterm delivery in Sweden was approximately 6% during 1973 to 2001.^21^ The 2× to 3× higher prevalence of preterm delivery we reported among women treated with coronary artery stenting indirectly supports the association between a history preterm delivery and future CAD. The increased risk of CAD observed in women with a history of spontaneous preterm delivery has been attributed to worse CVD risk factors, inflammation, and specific endothelial dysfunction.^22^ All these factors are also potentially involved in the pathological process leading to MACE in the secondary prevention setting. Women with a history of early preterm delivery have higher risk of developing CVD than those with a history of later preterm delivery.^23^ However, in this study of long-term outcomes following treatment with coronary artery stenting, we did not observe a similar dose-effect association with the outcome. In contrast, we found the risk of MACE in women with a history of early preterm delivery was lower than that of women with late preterm delivery. While the estimate for early preterm delivery was close to 1, its 95% CI still included the estimate for our main result (a 19% increase in HR). Understanding the extent to which the difference by preterm delivery subgroup was observed by chance, or represents an actual difference in risk, requires similar studies in other patient cohorts. In this study, we accounted for several important periprocedural risk factors of MACE in our analyses. In addition, the roughly similar risk estimates observed for all-cause mortality by late and early preterm delivery history, respectively, indirectly suggest that there was also no major difference in severe comorbidity at the time of stenting between the 2 groups.

### Clinical relevance

The association we report between preterm delivery and MACE is modest but clinically relevant. Identifying relevant aspects of a women’s reproductive history is already recommended in the primary prevention of heart diseases.^24^ Our results suggest that a woman’s pregnancy complication history might be relevant also in the secondary prevention setting. The extent to which the increased risk of MACE following coronary artery stenting in women with a history of preterm delivery can be lowered by existing secondary prevention strategies warrants further studies. Our results further underscores that a woman’s pregnancy complication history is not only relevant to obstetrician-gynecologists but warrants wider attention among clinicians. Furthermore, our results indicate that immediate access to a patient’s whole medical history, eg, through fully integrated medical records, might constitute a step forward toward improved acute cardiovascular care for women.

### Strengths and limitations

The main strength of the study is the comprehensive nationwide sample of women receiving coronary artery stents. Extensive information on women’s reproductive history collected over decades, together with available data on several covariates, resulted in a comprehensive sample with few exclusions. The coronary stenting outcome data have been collected in a well-known and established registry.^25^^,^^26^ Another strength is our use of multiple imputations to address missingness for some predictors used in the statistical analyses, with corresponding complete case analyses showing very similar results. In addition, the proportion of missing data for the imputed variables was small, and only a small proportion of included women had any missing data. However, this study also had some limitations. As the collection of data on deliveries started in 1973, cardiac care has generally improved during the studied time period of 2006 to 2017, and age is an important factor to consider for the prognosis following coronary artery stenting, we only included women aged 65 years or younger. Furthermore, women who experienced their first PCI prior to 2006 were excluded from the analysis due to the start date of complete variable collection in the registry. Pregnancy dating with obstetric ultrasound was not used clinically in Sweden until the 1970s, and at that time, not all women were routinely offered an ultrasonography. Therefore, not all pregnancies included in our study are dated using ultrasound, and the pregnancy dating (gestational week) may be less certain for women who gave birth in the earliest years included in the study. However, this potential misclassification should, at most, dilute our results. Given that preterm delivery history status is not considered in any relevant guidelines of acute cardiac care, it was likely not typically considered in the care of women included in this study. Thus, we consider the resulting risk of bias on treatment decisions to be negligible. Lastly, it should be noted that the relative ethnic homogeneity of the study sample potentially affects the generalization of the results to other populations.

## Conclusions

A history of preterm delivery was associated with worse prognosis following the first coronary artery stenting in women aged ≤65 years. Considering patients’ preterm delivery history might improve the acute cardiac care of women and facilitate secondary prevention.

PERSPECTIVES **COMPETENCY IN MEDICAL KNOWLEDGE:** Preterm delivery is a risk factor for future CAD, and women with a history of preterm delivery are more likely to experience a cardiovascular-related death than other women. A history of preterm delivery may be a prognostic marker of worse outcomes following coronary artery stenting. Women with a history of preterm delivery should be made aware of their future risk of cardiovascular disease in both the primary and secondary prevention settings.

**TRANSLATIONAL OUTLOOK 1:** The etiological mechanism by which women with a history of preterm delivery have higher risk of MACE following coronary artery stenting needs to be further studied.

**TRANSLATIONAL OUTLOOK 2:** The extent to which the increased risk of MACE following coronary artery stenting in women with a history of preterm delivery can be lowered by existing secondary prevention strategies should be characterized.

## Funding support and author disclosures

This work was supported by grants awarded to Simon Timpka from the 10.13039/501100004359Swedish Research Council (2019-02082); The 10.13039/501100003793Swedish Heart-Lung Foundation (20180312); public research support via the Faculty of Medicine at 10.13039/501100003252Lund University (ALF [Avtal om Läkarutbildning och Forskning]: YF-ALF, ALF project); The 10.13039/501100007687Swedish Society of Medicine (SLS-885331); The 10.13039/100008738Jeansson Foundations, Stockholm, Sweden; and 10.13039/100007435Åke Wiberg Foundation, Stockholm, Sweden. The authors have reported that they have no relationships relevant to the contents of this paper to disclose.
