# Supplementary material for: Long-Term Outcome Following Coronary Artery Stenting by History of Preterm Delivery
Source: JACC Adv. 2022 Nov 30;1(5):100142. doi: 10.1016/j.jacadv.2022.100142 (PMC11198654; doi:10.1016/j.jacadv.2022.100142)
Supplement: Supplemental Tables S1-S7 [file mmc1.docx]

| **Supplemental Table 1.** Definition of major cardiovascular adverse events (MACE) | | | | |
| --- | --- | --- | --- | --- |
|  |  |  |  |  |
| **Cardiovascular events** | **ICD 10** | **ICD 9** | **ICD 8** |  |
| Acute myocardial infarction | I21 | 410 | 410 |  |
| Subsequent myocardial infarction | I22 | 411 | 411 |  |
| Unstable or unspecified angina | I20.0  I20.9 | 413 | 413 |  |
| Cerebral infarction | I63 | 434 | 434 |  |
| Stroke, not specified as hemorrhage or infarction | I64 | 436 | 436 |  |
|  |  |  |  |  |
|  |  |  |  |  |
| **Cardiovascular deaths** | **ICD 10** | **ICD 9** | **ICD 8** |  |
| Unstable or unspecified angina | I20.0  I20.9 | 413 | 413 |  |
| Acute myocardial infarction | I21 | 410 | 410 |  |
| Subsequent myocardial infarction | I22 | 411 | 411 |  |
| Chronic ischemic heart disease | I25.0  I25.1  I25.2  I25.5  I25.6  I25.8  I25.9 | 412X  414A  414W  414X  429C | 410  412  414  429  4589 |  |
| Cardiac arrest | I46 | 427F | 427 |  |
| Sudden death, cause unknown | R96  R98  R99 | 798B  798C  798X  799W | 7825  795  796 |  |
| Atrial fibrillation and flutter | I48 | 427D | 4279 |  |
| Ventricular fibrillation and flutter | I49.0 | 427E | 4279 |  |
| Heart failure | I50 | 428  429X | 7824  428  429 |  |
| Cerebral infarction | I63 | 434 | 434 |  |

*Patients with >1 of the chosen ICD diagnoses as one of their first three diagnoses during a hospital stay at >30 days from PCI, were considered to have an event.
ICD, International Classification of diseases; PCI, percutaneous coronary intervention

| **Supplemental Table 2.** MACE following coronary artery stenting by preterm delivery, complete case (N=5,473) | | | | | | | | |
| --- | --- | --- | --- | --- | --- | --- | --- | --- |
|  |  | |  | | **Subgroups of preterm delivery** | | | |
|  |  | |  | | (n=905) | | | |
| **Preterm delivery history** | **No preterm delivery** | | **Ever preterm delivery** | | **Late preterm delivery** | | **Early preterm delivery** | |
| events / person years | 897 / 20,096 |  | 213 / 3,692 | | 154 / 2,411 | | 59 / 1,281 | |
|  |  |  | **HR (95% CI)** | ***p*** | **HR (95% CI)** | ***p*** | **HR (95% CI)** | ***p*** |
| Model I | 1 (reference) |  | 1.24 (1.07-1.44) | 0.005 | 1.37 (1.15-1.62) | <0.001 | 1.00 (0.77-1.31) | 0.99 |
| Model II | 1 (reference) |  | 1.23 (1.06-1.44) | 0.006 | 1.35 (1.14-1.61) | 0.001 | 1.00 (0.77-1.31) | 1.00 |
| Model III | 1 (reference) |  | 1.18 (1.01-1.38) | 0.03 | 1.31 (1.11-1.56) | 0.002 | 0.93 (0.71-1.21) | 0.59 |
| Model I includes history of preterm delivery; age at index PCI [continuous]  Model II additionally includes procedure type [PCI, PCI ad hoc]; indication for PCI [STEMI, NSTEMI, unstable coronary artery disease,  stable coronary artery disease, other]; year of procedure [2006-2009, 2010-2013, 2014-2017]; number of vessels treated [1, >2];  number of stents [1, 2, >3]; multiple vessel disease [yes/no]; drug eluting stent [yes/no]; left main stem treated or left anterior descending  artery treated [yes/no]; right coronary artery treated [yes/no]; left circumflex coronary artery treated [yes/no]; any other vessel treated [yes/no]  Model III additionally includes diabetes [yes/no]; smoking [never smoker, ex-smoker, current smoker]; hypertension [yes/no];  dyslipidaemia [yes/no]; prior MI [yes/no]  Results from cox proportional hazards regression, complete case analysis. Median follow-up time 3.72 years (interquartile range, 1.47 to 6.74 years).  * CI, confidence interval; HR, hazard ratio; MACE, major adverse cardiovascular events; MI, myocardial infarction; NSTEMI, non-ST-elevation myocardial infarction; PCI, percutaneous coronary intervention; STEMI, ST-elevation myocardial infarction | | | | | | | | |

| **Supplemental Table 3.** MACE following coronary artery stenting by preterm delivery, HDP excluded (N=4,975) | | | | | | | | |
| --- | --- | --- | --- | --- | --- | --- | --- | --- |
|  |  | |  | | **Subgroups of preterm delivery** | | | |
|  |  | |  | | (n=719) | | | |
| **Preterm delivery history** | **No preterm delivery** | | **Ever preterm delivery** | | **Late preterm delivery** | | **Early preterm delivery** | |
| events / person years | 840 / 18,653 |  | 171 / 2,851 | | 127 / 1,953 | | 44 / 897 | |
|  |  |  | **HR (95% CI)** | ***p*** | **HR (95% CI)** | ***p*** | **HR (95% CI)** | ***p*** |
| Model I | 1 (reference) |  | 1.27 (1.08-1.50) | 0.004 | 1.37 (1.14-1.65) | 0.001 | 1.06 (0.78-1.43) | 0.71 |
| Model II | 1 (reference) |  | 1.28 (1.09-1.51) | 0.003 | 1.37 (1.14-1.65) | 0.001 | 1.07 (0.79-1.46) | 0.65 |
| Model III | 1 (reference) |  | 1.21 (1.02-1.43) | 0.03 | 1.31 (1.09-1.59) | 0.005 | 0.98 (0.72-1.34) | 0.91 |
| Model I includes history of preterm delivery; age at index PCI [continuous]  Model II additionally includes procedure type [PCI, PCI ad hoc]; indication for PCI [STEMI, NSTEMI, unstable coronary artery disease,  stable coronary artery disease, other]; year of procedure [2006-2009, 2010-2013, 2014-2017]; number of vessels treated [1, >2];  number of stents [1, 2, >3]; multiple vessel disease [yes/no]; drug eluting stent [yes/no]; left main stem treated or left anterior descending  artery treated [yes/no]; right coronary artery treated [yes/no]; left circumflex coronary artery treated [yes/no]; any other vessel treated [yes/no]  Model III additionally includes diabetes [yes/no]; smoking [never smoker, ex-smoker, current smoker]; hypertension [yes/no];  dyslipidaemia [yes/no]; prior MI [yes/no]  Results from cox proportional hazards regression, multiple imputation analysis. Analysis restricted to women with no history of hypertensive disorder of pregnancy. Median follow-up time 3.65 years (interquartile range, 1.44 to 6.74 years).  * CI, confidence interval; HDP, hypertensive disorder of pregnancy; HR, hazard ratio; MACE, major adverse cardiovascular events; MI, myocardial infarction; NSTEMI, non-ST-elevation myocardial infarction; PCI, percutaneous coronary intervention; STEMI, ST-elevation myocardial infarction | | | | | | | | |

| **Supplemental Table 4.** MACE following coronary artery stenting by preterm delivery, diabetes excluded (N=4,845) | | | | | | | | |
| --- | --- | --- | --- | --- | --- | --- | --- | --- |
|  |  | |  | | **Subgroups of preterm delivery** | | | |
|  |  | |  | | (n=734) | | | |
| **Preterm delivery history** | **No preterm delivery** | | **Ever preterm delivery** | | **Late preterm delivery** | | **Early preterm delivery** | |
| events / person years | 761 / 18,368 |  | 161 / 3,028 | | 114 / 2,016 | | 47 / 1,011 | |
|  |  |  | **HR (95% CI)** | ***p*** | **HR (95% CI)** | ***p*** | **HR (95% CI)** | ***p*** |
| Model I | 1 (reference) |  | 1.23 (1.04-1.46) | 0.02 | 1.30 (1.07-1.59) | 0.008 | 1.09 (0.81-1.46) | 0.59 |
| Model II | 1 (reference) |  | 1.25 (1.05-1.48) | 0.01 | 1.31 (1.08-1.60) | 0.007 | 1.12 (0.83-1.51) | 0.45 |
| Model III | 1 (reference) |  | 1.24 (1.05-1.48) | 0.01 | 1.30 (1.07-1.59) | 0.009 | 1.12 (0.83-1.50) | 0.46 |
| Model I includes history of preterm delivery; age at index PCI [continuous]  Model II additionally includes procedure type [PCI, PCI ad hoc]; indication for PCI [STEMI, NSTEMI, unstable coronary artery disease,  stable coronary artery disease, other]; year of procedure [2006-2009, 2010-2013, 2014-2017]; number of vessels treated [1, >2];  number of stents [1, 2, >3]; multiple vessel disease [yes/no]; drug eluting stent [yes/no]; left main stem treated or left anterior descending  artery treated [yes/no]; right coronary artery treated [yes/no]; left circumflex coronary artery treated [yes/no]; any other vessel treated [yes/no]  Model III additionally includes smoking [never smoker, ex-smoker, current smoker]; hypertension [yes/no]; dyslipidaemia [yes/no]; prior MI [yes/no]  Results from cox proportional hazards regression, multiple imputation analysis. Median follow-up time 3.79 years (interquartile range, 1.50 to 6.89 years). Analysis restricted to women without diabetes mellitus.  *CI, confidence interval; HR, hazard ratio; MACE, major adverse cardiovascular events; MI, myocardial infarction; NSTEMI, non-ST-elevation myocardial infarction; PCI, percutaneous coronary intervention; STEMI, ST-elevation myocardial infarction | | | | | | | | |

| **Supplemental Table 5.** Long-term mortality following coronary artery stenting by preterm delivery, complete case (N=5,473) | | | | | | | | |
| --- | --- | --- | --- | --- | --- | --- | --- | --- |
|  |  | |  | | **Subgroups of preterm delivery** | | | |
|  |  | |  | | (n=905) | | | |
| **Preterm delivery history** | **No preterm delivery** | | **Ever preterm delivery** | | **Late preterm delivery** | | **Early preterm delivery** | |
| events / person years | 178 / 24,164 |  | 53 / 4,603 | | 36 / 3,079 | | 17 / 1,524 | |
|  |  |  | **HR (95% CI)** | ***p*** | **HR (95% CI)** | ***p*** | **HR (95% CI)** | ***p*** |
| Model I | 1 (reference) |  | 1.72 (1.26-2.34) | 0.001 | 1.68 (1.18-2.41) | 0.004 | 1.79 (1.09-2.96) | 0.02 |
| Model II | 1 (reference) |  | 1.71 (1.25-2.33) | 0.001 | 1.68 (1.17-2.40) | 0.005 | 1.78 (1.08-2.94) | 0.03 |
| Model III | 1 (reference) |  | 1.48 (1.08-2.02) | 0.01 | 1.47 (1.03-2.12) | 0.04 | 1.49 (0.90-2.48) | 0.12 |
| Model I includes history of preterm delivery; age at index PCI [continuous]  Model II additionally includes procedure type [PCI, PCI ad hoc]; indication for PCI [STEMI, NSTEMI, unstable coronary artery disease,  stable coronary artery disease, other]; year of procedure [2006-2009, 2010-2013, 2014-2017]; number of vessels treated [1, >2];  number of stents [1, 2, >3]; multiple vessel disease [yes/no]; drug eluting stent [yes/no]; left main stem treated or left anterior descending  artery treated [yes/no]; right coronary artery treated [yes/no]; left circumflex coronary artery treated [yes/no]; any other vessel treated [yes/no]  Model III additionally includes diabetes [yes/no]; smoking [never smoker, ex-smoker, current smoker]; hypertension [yes/no];  dyslipidaemia [yes/no]; prior MI [yes/no]  Results from cox proportional hazards regression, complete case analysis. Median follow-up time 4.91 years (interquartile range, 2.42 to 7.89 years).  *CI, confidence interval; HR, hazard ratio; MI, myocardial infarction; NSTEMI, non-ST-elevation myocardial infarction;  PCI, percutaneous coronary intervention; STEMI, ST-elevation myocardial infarction | | | | | | | | |

| **Supplemental Table 6.** Long-term mortality following coronary artery stenting by preterm delivery, HDP excluded (N=4,975) | | | | | | | | |
| --- | --- | --- | --- | --- | --- | --- | --- | --- |
|  |  | |  | | **Subgroups of preterm delivery** | | | |
|  |  | |  | | (n=719) | | | |
| **Preterm delivery history** | **No preterm delivery** | | **Ever preterm delivery** | | **Late preterm delivery** | | **Early preterm delivery** | |
| events / person years | 177 / 22,471 |  | 51 / 3,566 | | 35 / 2,513 | | 16 / 1,052 | |
|  |  |  | **HR (95% CI)** | ***p*** | **HR (95% CI)** | ***p*** | **HR (95% CI)** | ***p*** |
| Model I | 1 (reference) |  | 1.94 (1.42-2.64) | <0.001 | 1.85 (1.29-2.66) | 0.001 | 2.16 (1.29-3.60) | 0.003 |
| Model II | 1 (reference) |  | 1.92 (1.40-2.62) | <0.001 | 1.84 (1.28-2.66) | 0.001 | 2.10 (1.25-3.52) | 0.005 |
| Model III | 1 (reference) |  | 1.58 (1.15-2.18) | 0.005 | 1.56 (1.08-2.25) | 0.02 | 1.65 (0.97-2.79) | 0.06 |
| Model I includes history of preterm delivery; age at index PCI [continuous]  Model II additionally includes procedure type [PCI, PCI ad hoc]; indication for PCI [STEMI, NSTEMI, unstable coronary artery disease,  stable coronary artery disease, other]; year of procedure [2006-2009, 2010-2013, 2014-2017]; number of vessels treated [1, >2];  number of stents [1, 2, >3]; multiple vessel disease [yes/no]; drug eluting stent [yes/no]; left main stem treated or left anterior descending  artery treated [yes/no]; right coronary artery treated [yes/no]; left circumflex coronary artery treated [yes/no]; any other vessel treated [yes/no]  Model III additionally includes diabetes [yes/no]; smoking [never smoker, ex-smoker, current smoker]; hypertension [yes/no];  dyslipidaemia [yes/no]; prior MI [yes/no]  Results from cox proportional hazards regression, multiple imputation analysis. Analysis restricted to women with no history of hypertensive disorder of pregnancy. Median follow-up time 4.85 years (interquartile range, 2.41 to 7.89 years).  *CI, confidence interval; HDP, hypertensive disorder of pregnancy; HR, hazard ratio; MACE, major adverse cardiovascular events; MI, myocardial infarction; NSTEMI, non-ST-elevation myocardial infarction; PCI, percutaneous coronary intervention; STEMI, ST-elevation myocardial infarction | | | | | | | | |

| **Supplemental Table 7.** Long-term mortality following coronary artery stenting by history of preterm delivery, diabetes excluded (N=4,845) | | | | | | | | |
| --- | --- | --- | --- | --- | --- | --- | --- | --- |
|  |  | |  | | **Subgroups of preterm delivery** | | | |
|  |  | |  | | (n=734) | | | |
| **Preterm delivery history** | **No preterm delivery** | | **Ever preterm delivery** | | **Late preterm delivery** | | **Early preterm delivery** | |
| events / person years | 139 / 21,944 |  | 37 / 3,726 | | 24 / 2,517 | | 13 / 1,209 | |
|  |  |  | **HR (95% CI)** | ***p*** | **HR (95% CI)** | ***p*** | **HR (95% CI)** | ***p*** |
| Model I | 1 (reference) |  | 1.68 (1.17-2.42) | 0.005 | 1.58 (1.02-2.43) | 0.04 | 1.92 (1.10-3.40) | 0.02 |
| Model II | 1 (reference) |  | 1.65 (1.14-2.37) | 0.007 | 1.57 (1.02-2.43) | 0.04 | 1.81 (1.02-3.20) | 0.04 |
| Model III | 1 (reference) |  | 1.49 (1.04-2.16) | 0.03 | 1.39 (0.88-2.16) | 0.14 | 1.72 (0.97-3.06) | 0.06 |
| Model I includes history of preterm delivery; age at index PCI [continuous]  Model II additionally includes procedure type [PCI, PCI ad hoc]; indication for PCI [STEMI, NSTEMI, unstable coronary artery disease,  stable coronary artery disease, other]; year of procedure [2006-2009, 2010-2013, 2014-2017]; number of vessels treated [1, >2];  number of stents [1, 2, >3]; multiple vessel disease [yes/no]; drug eluting stent [yes/no]; left main stem treated or left anterior descending  artery treated [yes/no]; right coronary artery treated [yes/no]; left circumflex coronary artery treated [yes/no]; any other vessel treated [yes/no]  Model III additionally includes smoking [never smoker, ex-smoker, current smoker]; hypertension [yes/no]; dyslipidaemia [yes/no]; prior MI [yes/no]  Results from cox proportional hazards regression, multiple imputation analysis. Median follow-up time 4.93 years (interquartile range, 2.43 to 8.02 years).  Analysis restricted to women without diabetes mellitus  *CI, confidence interval; HR, hazard ratio; MACE, major adverse cardiovascular events; MI, myocardial infarction; NSTEMI, non-ST-elevation myocardial infarction; PCI, percutaneous coronary intervention; STEMI, ST-elevation myocardial infarction | | | | | | | | |
